# miRDB: an online database for prediction of functional microRNA targets

**DOI:** 10.1093/nar/gkz757

**Published:** 2019-08-31

**Authors:** Yuhao Chen, Xiaowei Wang

**Affiliations:** 1 Department of Radiation Oncology, Washington University School of Medicine, St Louis, MO, USA; 2 Department of Electrical and Systems Engineering, Washington University in St Louis, St Louis, MO, USA

## Abstract

MicroRNAs (miRNAs) are small noncoding RNAs that act as master regulators in many biological processes. miRNAs function mainly by downregulating the expression of their gene targets. Thus, accurate prediction of miRNA targets is critical for characterization of miRNA functions. To this end, we have developed an online database, miRDB, for miRNA target prediction and functional annotations. Recently, we have performed major updates for miRDB. Specifically, by employing an improved algorithm for miRNA target prediction, we now present updated transcriptome-wide target prediction data in miRDB, including 3.5 million predicted targets regulated by 7000 miRNAs in five species. Further, we have implemented the new prediction algorithm into a web server, allowing custom target prediction with user-provided sequences. Another new database feature is the prediction of cell-specific miRNA targets. miRDB now hosts the expression profiles of over 1000 cell lines and presents target prediction data that are tailored for specific cell models. At last, a new web query interface has been added to miRDB for prediction of miRNA functions by integrative analysis of target prediction and Gene Ontology data. All data in miRDB are freely accessible at http://mirdb.org.

## INTRODUCTION

MicroRNAs (miRNAs) are small noncoding RNAs that regulate many biological processes (1,2). About 2000 human miRNAs have been reported in miRBase (3). Computational and experimental analyses indicate that most known protein-coding genes are regulated by miRNAs at both post-transcriptional and translational levels (4–6). miRNAs function mainly by downregulating the expression of their gene targets. Thus, accurate prediction of miRNA targets is critical for characterizing miRNA functions. At present, reliable identification of miRNA targets is still a major challenge, and many researchers choose to use computational tools to predict candidate gene targets for further experimental validation. To facilitate the process of candidate target selection, we have previously developed an online database, miRDB, for miRNA target prediction and functional annotations (7,8). Here, we present major updates to miRDB, most noticeably the presentation of updated target prediction data based on an improved computational algorithm. Other new features include prediction of miRNA targets in specific cell models, and prediction of miRNA-regulated biological processes by integrative analysis of target prediction and Gene Ontology (GO) data. The web server interface of miRDB has been updated to present these new database features. All data in miRDB are freely accessible at http://mirdb.org.

## DATABASE UPDATES

### Presentation of updated target prediction data

**Figure 1.**
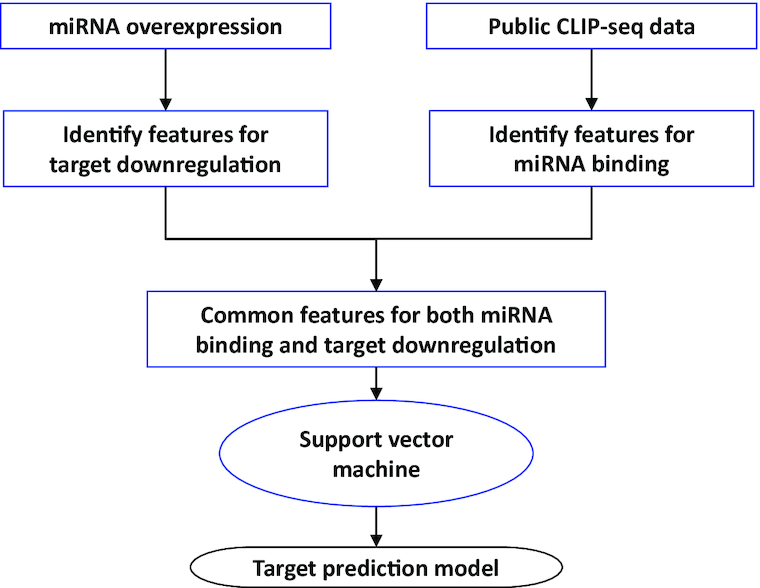
Overview of the updated miRNA target prediction algorithm, MirTarget. Training data were derived from both miRNA binding and target expression data. MirTarget was trained under an SVM machine learning framework.

We have recently developed an improved computational model for miRNA target prediction. Details of this prediction model have been described in our recent publication (9). The workflow of the model development process is presented in Figure 1. One unique aspect of the algorithm development process is the quality as well as the comprehensiveness of the training data. Specifically, we have performed a large-scale RNA-seq study to globally profile the impact on target expression by individual miRNAs. To our knowledge, our RNA-seq profiling dataset, consisting of 1.5 billion reads from 52 RNA samples, represents the largest of its kind for miRNA target analysis. By focusing on transcripts that are downregulated by miRNA overexpression, we were able to discover and further quantify miRNA targeting features that are characteristic of target downregulation. On the other hand, we also analyzed public CLIP-ligation data (10,11) to identify paired miRNA/target transcripts that reside in the same miRISC complex. In this way, we were able to identify features that are associated with miRNA target binding. Next, by integratively analyzing both miRNA binding and target downregulation data, we were able to identify significant targeting features that are common for both processes. At last, a support vector machine (SVM) model, **MirTarget**, was trained with the identified features for miRNA target prediction. Comparative analysis using independent datasets indicates that MirTarget has improved performance over other existing prediction algorithms (9).

With MirTarget, we performed transcriptome-wide miRNA target prediction for five species: human, mouse, rat, dog and chicken. Specifically, the miRNA sequences were downloaded from miRBase version 22 (3); target transcript sequences were retrieved from the NCBI RefSeq database (12) and further parsed with BioPerl to extract the 3’-UTR sequences. Unlike many existing algorithms, evolutionary conservation of the target binding site is not a required feature for miRNA target prediction with MirTarget. In this way, both conserved and nonconserved targets can be predicted by MirTarget. For each candidate target site, MirTarget generates a probability score as computed by the underlying SVM modeling tool. This score reflects statistical confidence of the prediction results. If a transcript contains multiple candidate target sites, individual site scores are combined to compute a final score for the entire transcript, as described in detail in (9). MirTarget prediction scores are in the range of 0–100, and candidate transcripts with scores ≥ 50 are presented as predicted miRNA targets in miRDB. In total, 3.5 million gene targets were predicted to be regulated by 7000 miRNAs across five species in the current version of miRDB (Version 6.0, Table 1). In comparison, 2.1 million gene targets and 6700 miRNAs were included in the previous version (Version 5.0). On average, there are 497 gene targets per miRNA across the five species, an increase of 58% from the previous version. The significant increase in gene target number is mainly a result of newly implemented MirTarget features such as integrative analysis of multiple miRNA seed types in a single prediction model and more comprehensive assessment of cross-species conservation of the seed binding sites. Specifically for humans, the number of predicted targets per miRNA is 606, which is significantly higher than other species. This likely reflects the relatively rich annotations of the human transcriptome as compared to other transcriptomes.

**Table 1.** Summary statistics of miRDB target prediction data

**Species**	**Mature miRNAs**	**Total gene targets**	**Unique gene targets**
Human	2656	1 610 510	29 161
Mouse	1978	986 416	22 499
Rat	764	187 303	12 612
Dog	453	170 435	16 710
Chicken	1235	565 220	21 577
**Total**	**7086**	**3 519 884**	**102 559**
*Previous Version (Version 5.0)*			
Human	2588	947 941	17 925
Mouse	1912	634 009	18 639
Rat	764	179 539	15 489
Dog	453	128 703	13 150
Chicken	992	214 816	12 911
**Total**	**6709**	**2 105 008**	**78 114**

miRDB presents a flexible web server interface for miRNA target retrieval. The default query form, the Target Search page, allows the users to retrieve target prediction data for one specific miRNA or gene target at a time. In addition, an advanced query form, the Target Mining page, enables the search for multiple miRNAs or gene targets at the same time. The Target Mining page also presents additional search filters to enable various combinations of search strategies based on user preference for miRNA and target selection.

### Custom target prediction by implementing MirTarget into a web server

We performed a major update on the custom prediction function of miRDB by implementing the new MirTarget algorithm. miRDB allows the users to provide custom miRNA or gene target sequences for transcriptome-wide prediction of gene targets or miRNA regulators in one of the five species: human, mouse, rat, dog or chicken. The custom sequence length is in the range of 17–30 nt for miRNA and 100–30 000 nt for gene target. The users should first select the species and search type (miRNA or gene target), and then input their custom sequence. Then, the Perl script implementing the MirTarget algorithm takes the web form inputs and starts the target prediction process. Two precompiled sequence files are used to predict potential miRNA/target pairs: one contains the 3’-UTR sequences from all known genes in the five species and the other one contains the sequences of species-specific miRNAs, as collated from miRBase version 22.

The target prediction process is as follows. First, the web server script collects the miRNA or candidate sequence from the web form. If the users input a custom miRNA sequence, the server script will import all 3’-UTR sequences from the selected species for target prediction; on the other hand, if the users input a candidate target sequence, all miRNA sequences from the selected species will be imported. Next, for every miRNA/candidate target pair, the server script scans for miRNA seed binding sites and generates targeting features for MirTarget prediction. The prediction data are presented as an annotation table for all miRNA/candidate target pairs, including target prediction scores and the miRNA/target sequences.

The prediction results are sorted in descending order as ranked by the target score. Then, the web server script imports the sorted results for web presentation, including target rank, target prediction score, miRNA name, target gene symbol and description. Additionally, the users have the option to review the details of the prediction result for every miRNA/target pair, including miRNA sequence and target sequence with highlighted miRNA seed binding positions. Depending on the number of predicted targets for the input miRNA, the whole prediction process typically completes in about 30–60 s.

### Target expression profiles in specific cell models

**Figure 2.**
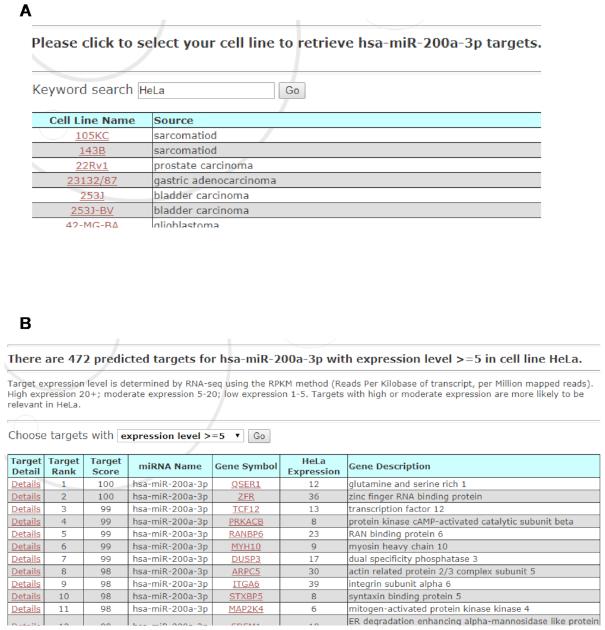
miRDB target expression analysis. miRDB hosts the expression profiles of over 1000 cell lines. (**A**) A screenshot for selection of specific cell models. (**B**) A screenshot for integrative presentation of both target prediction and expression data for the selected cell model.

By default, miRDB presents miRNA target prediction data for all known genes in the genome. However, not all potential miRNA targets are functionally relevant in a given cell. Thus, researchers often need to perform target analysis in the context of specific cell models. To facilitate the selection of cell-specific miRNA targets, miRDB presents a Target Expression page, which enables the users to combine target prediction data with target expression profiles from over 1000 cell lines (Figure 2A). Specifically, we downloaded RNA-seq gene expression profiling data from two large-scale transcriptome studies (13,14) that were deposited in Expression Atlas (15). Combined together, these studies have profiled RNA expression in 1178 cell lines by RNA-seq analysis. Gene expression levels are represented as normalized RPKM read counts (Reads Per Kilobase of transcript per Million mapped reads). Based on the expression values, we defined four gene groups: high expression (RPKM > 20), moderate expression (RPKM 5–20), low expression (RPKM 1–5) and no detectable expression (RPKM < 1). On average, there are about 11 000 genes with detectable expression per cell line. Gene targets with high or moderate expression in a specific cell model are more likely to be functionally impacted by miRNA regulation. As shown in Figure 2B, the users may further limit target selection by defining a desired gene expression threshold. The expression level of each gene target is presented together with MirTarget prediction score. By integrating target prediction and expression data, the users can quickly identify cell-specific targets for further experimental validation.

### Prediction of miRNA functions by target ontology analysis

**Figure 3.**
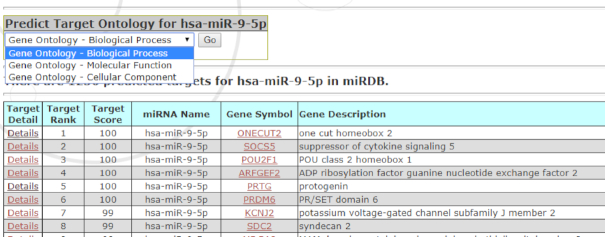
A screenshot for miRDB target ontology analysis. miRNA target prediction data and GO data were integratively analyzed to predict miRNA functions.

The function of a miRNA is defined by its gene targets. Thus, biological pathways regulated by miRNAs can be inferred by target analysis. However, as one miRNA can potentially regulate hundreds of gene targets, it is a challenge to reliably identify significant pathways impacted by miRNA regulation. One popular approach for miRNA functional prediction is to perform target enrichment analysis, i.e. identifying pathways or functional categories that are statistically enriched in miRNA targets. To this end, we have implemented a new web interface for target ontology analysis. As presented in the Target Ontology page, the users may first retrieve all predicted targets for a specific miRNA of interest, and then directly submit the target list for GO enrichment analysis (Figure 3). The GO enrichment analysis is performed by employing the PANTHER web server engine (16) using up-to-date GO terms (17). By providing a new web query interface, miRDB integrates both target prediction and GO enrichment analyses, and presents a streamlined pipeline for prediction of miRNA functions.

In summary, we have performed major updates on miRDB, including implementation of a new miRNA target prediction algorithm as well as presentation of new database features for prediction of miRNA functions. By combining miRNA target prediction data with other biological data such as cell-specific expression profiles or GO annotations, we expect these new miRDB features to be helpful for researchers to quickly identify relevant miRNA functions in specific experimental systems. In the future, we will continue to make improvements to the target prediction algorithm as well as integrate more heterogeneous types of data in miRDB for flexible analysis of miRNA functions in various experimental settings.

## DATA AVAILABILITY

All data in miRDB are freely accessible at http://mirdb.org.

## References

[1] Ambros V. The functions of animal microRNAs. Nature. 2004; 431:350–355. 15372042 10.1038/nature02871

[2] Miska E.A. How microRNAs control cell division, differentiation and death. Curr. Opin. Genet. Dev. 2005; 15:563–568. 16099643 10.1016/j.gde.2005.08.005

[3] Kozomara A., Birgaoanu M., Griffiths-Jones S. miRBase: from microRNA sequences to function. Nucleic Acids Res. 2019; 47:D155–D162. 30423142 10.1093/nar/gky1141 PMC6323917

[4] Lim L.P., Lau N.C., Garrett-Engele P., Grimson A., Schelter J.M., Castle J., Bartel D.P., Linsley P.S., Johnson J.M. Microarray analysis shows that some microRNAs downregulate large numbers of target mRNAs. Nature. 2005; 433:769–773. 15685193 10.1038/nature03315

[5] Baek D., Villen J., Shin C., Camargo F.D., Gygi S.P., Bartel D.P. The impact of microRNAs on protein output. Nature. 2008; 455:64–71. 18668037 10.1038/nature07242 PMC2745094

[6] Selbach M., Schwanhausser B., Thierfelder N., Fang Z., Khanin R., Rajewsky N. Widespread changes in protein synthesis induced by microRNAs. Nature. 2008; 455:58–63. 18668040 10.1038/nature07228

[7] Wong N., Wang X. miRDB: an online resource for microRNA target prediction and functional annotations. Nucleic Acids Res. 2015; 43:D146–D152. 25378301 10.1093/nar/gku1104 PMC4383922

[8] Wang X. miRDB: a microRNA target prediction and functional annotation database with a wiki interface. RNA. 2008; 14:1012–1017. 18426918 10.1261/rna.965408 PMC2390791

[9] Liu W., Wang X. Prediction of functional microRNA targets by integrative modeling of microRNA binding and target expression data. Genome Biol. 2019; 20:18. 30670076 10.1186/s13059-019-1629-z PMC6341724

[10] Helwak A., Kudla G., Dudnakova T., Tollervey D. Mapping the human miRNA interactome by CLASH reveals frequent noncanonical binding. Cell. 2013; 153:654–665. 23622248 10.1016/j.cell.2013.03.043 PMC3650559

[11] Grosswendt S., Filipchyk A., Manzano M., Klironomos F., Schilling M., Herzog M., Gottwein E., Rajewsky N. Unambiguous identification of miRNA:target site interactions by different types of ligation reactions. Mol. Cell. 2014; 54:1042–1054. 24857550 10.1016/j.molcel.2014.03.049 PMC4181535

[12] O’Leary N.A., Wright M.W., Brister J.R., Ciufo S., Haddad D., McVeigh R., Rajput B., Robbertse B., Smith-White B., Ako-Adjei D. et al. Reference sequence (RefSeq) database at NCBI: current status, taxonomic expansion, and functional annotation. Nucleic Acids Res. 2016; 44:D733–D745. 26553804 10.1093/nar/gkv1189 PMC4702849

[13] Barretina J., Caponigro G., Stransky N., Venkatesan K., Margolin A.A., Kim S., Wilson C.J., Lehar J., Kryukov G.V., Sonkin D. et al. The Cancer Cell Line Encyclopedia enables predictive modelling of anticancer drug sensitivity. Nature. 2012; 483:603–607. 22460905 10.1038/nature11003 PMC3320027

[14] Klijn C., Durinck S., Stawiski E.W., Haverty P.M., Jiang Z., Liu H., Degenhardt J., Mayba O., Gnad F., Liu J. et al. A comprehensive transcriptional portrait of human cancer cell lines. Nat. Biotechnol. 2015; 33:306–312. 25485619 10.1038/nbt.3080

[15] Papatheodorou I., Fonseca N.A., Keays M., Tang Y.A., Barrera E., Bazant W., Burke M., Fullgrabe A., Fuentes A.M., George N. et al. Expression Atlas: gene and protein expression across multiple studies and organisms. Nucleic Acids Res. 2018; 46:D246–D251. 29165655 10.1093/nar/gkx1158 PMC5753389

[16] Mi H., Muruganujan A., Ebert D., Huang X., Thomas P.D. PANTHER version 14: more genomes, a new PANTHER GO-slim and improvements in enrichment analysis tools. Nucleic Acids Res. 2019; 47:D419–D426. 30407594 10.1093/nar/gky1038 PMC6323939

[17] The Gene Ontology Consortium The Gene Ontology Resource: 20 years and still GOing strong. Nucleic Acids Res. 2019; 47:D330–D338. 30395331 10.1093/nar/gky1055 PMC6323945

